# Circadian Rhythms and Mood Disorders: Are the Phenomena and Mechanisms Causally Related?

**DOI:** 10.3389/fpsyt.2015.00118

**Published:** 2015-08-24

**Authors:** William Bechtel

**Affiliations:** ^1^Department of Philosophy and Center for Circadian Biology, University of California San Diego, San Diego, CA, USA

**Keywords:** mood disorders, circadian rhythms, mechanistic explanations, causal relations between mechanisms

## Abstract

This paper reviews some of the compelling evidence of disrupted circadian rhythms in individuals with mood disorders (major depressive disorder, seasonal affective disorder, and bipolar disorder) and that treatments such as bright light, designed to alter circadian rhythms, are effective in treating these disorders. Neurotransmitters in brain regions implicated in mood regulation exhibit circadian rhythms. A mouse model originally employed to identify a circadian gene has proven a potent model for mania. While this evidence is suggestive of an etiological role for altered circadian rhythms in mood disorders, it is compatible with other explanations, including that disrupted circadian rhythms and mood disorders are effects of a common cause and that genes and proteins implicated in both simply have pleiotropic effects. In light of this, the paper advances a proposal as to what evidence would be needed to establish a direct causal link between disruption of circadian rhythms and mood disorders.

## Introduction

Much biological research over the past 200 years has proceeded by delineating individual phenomena and explaining each distinct phenomenon by characterizing the responsible mechanism (1). Discovering mechanisms involves localizing the phenomenon in a responsible system that is taken to be the mechanism and then decomposing that system structurally to discover its component parts and functionally to discover the operations those parts perform (2–4). To show that the parts and operations suffice for the phenomenon requires recomposing the mechanism by mentally rehearsing the operations or employing computational models. When the organization is non-sequential, and the operations, non-linear, computational modeling is often necessary to establish that the mechanism could generate the phenomenon (5). Overall, this has been a highly productive strategy. Much has been learnt about the mechanisms for various biological phenomena, but it has also succeeded in revealing the limitations of the approach. One important limitation is that the supposedly independent phenomena and mechanisms are not nearly as independent as initially thought.

Understanding the ways mechanisms are connected is turning out to be an important challenge in twenty-first century biology and medicine, in part because such connections afford useful ways of intervening on systems to control particular phenomena (e.g., to treat specific diseases). This paper explores the challenges in establishing that the mechanism advanced to explain the fact that one phenomenon is causally affected by that put forward to explain another. As in the project of advancing a philosophical analysis of mechanistic explanation, my aim is to ground an account of what is involved in establishing causal relations between mechanisms in the practice of scientific researchers. To do this, I focus on two biological phenomena in which important progress has been made in identifying mechanisms – circadian rhythms and moods – and engage in a detailed review of the research and the causal claims that have been advanced as to how the mechanisms relate to each other^1^.

As much of the research on mood has focused not on the moods of healthy individuals, but mood disorders such as major depressive disorder (MDD) and bipolar disorder (BD), and these have been related to disruptions of normal circadian rhythms, I will generally treat the related phenomena as mood disorders and disruptions of circadian rhythms. While making for a somewhat complex discussion, this does not pose a substantive problem since disrupted phenomena often serve to guide development of the understanding of the mechanism underlying the normally occurring phenomenon (6).

Progress in characterizing and understanding circadian rhythms and moods has largely stemmed from treating the two independently. But research in the later decades of the twentieth century also revealed connections between the two phenomena by showing that mood disorders are accompanied by disruptions of circadian rhythms. During the last two decades research on both circadian rhythms and mood disorders has increasingly focused on the molecular components of the two mechanisms. This research has revealed that molecular components of the circadian clock mechanism also play a role in mood disorders. This has raised the possibility of causal links between the mechanisms so that either disruptions in the circadian mechanism might be viewed as a cause of mood disorders, or mood disorders might be the cause of altered circadian rhythms. Of course the causality could also go in both directions, but my focus is on another alternative – that despite involving shared components, the two mechanisms are really independent and that what researchers are observing are effects of a common cause or pleotropic effects of common components.

My goal in this paper is not to argue for a particular stance on whether circadian rhythm disorders cause mood disorders; rather I will show how research has raised and addressed the question of possible connections between the two phenomena in the Section “Establishing a Relation between Circadian Rhythms and Mood Disorders” and between the two mechanisms in the Section “Research Implicating Molecular Components of the Circadian Clock in Mood Disorders.” I then explore whether a causal connection is supported by the evidence in the Section “How are the Mechanisms Related?” In particular, I am concerned with what it would take to establish a causal connection between these mechanisms. Focusing only on the relation between phenomena, the standard measure of independence is that each can be dissociated from the other. There is evidence that some features of mood disorders and circadian disruptions are independent, but the same is true of different features that are treated as aspects of just one phenomenon. The separateness or relatedness of the mechanisms is what ultimately will determine whether researchers judge the phenomena to be causally linked. Mechanisms comprise parts performing operations, which explains the focus on determining whether parts of the circadian mechanism are also implicated in mood phenomena. However, just knowing that two mechanisms employ the same type of part, even if it performs the same operation in each, does not causally link the two mechanisms. Two car engines may employ pistons, but that does not causally connect them. What is required is that the same individual part is a component of both mechanisms and that the parts of the first mechanism affect the second mechanism differently as the first mechanism is in different states. Only then can researchers relate activities in one mechanism to activities in another mechanism. In the case of circadian and mood mechanisms, one needs to show that a protein, for example, affects mood differently as a consequence of its role in different circadian states. This is a demanding standard that has not yet been realized. But before raising these skeptical worries about how circadian disruptions and mood disorders relate, I turn first to the evidence that has been invoked in relating them. As noted above, my objective is to show that the question of how to understand the relation between the generation of circadian rhythm disorders and mood disorders is a real concern for science, not purely an abstract philosophical concern. Subsequent sections, thus, provide a review of how claims about the relation between circadian rhythms and mood developed and the current state of attempts to evaluate whether a causal claim can be substantiated.

## Establishing a Relation between Circadian Rhythms and Mood Disorders

Reports of daily cycles of leaf folding in plants stem from ancient times and were first shown experimentally not to be a response to light when De Mairan (7) placed plants in a dark cupboard and observed that they continued to fold. Other examples of daily rhythms followed, such as, Wunderlich’s (8) demonstration that body temperature in humans oscillates by over 1°C/day. Although a variety of researchers tried to maintain that these were responses to external cues, by the time of the International Symposium on Biological Clocks in 1960, the recognition that these oscillations continue but with periods of only approximately 24 h (thus, *circa* + *dies*) when light, temperature, and other environmental cues are removed had established that these rhythms were generated endogenously. That fact, plus the ability of these oscillations to be entrained by light or other cues (referred to as *Zeitgebers*) and the determination that they were maintained with the same period at different temperatures, has come to characterize the phenomenon of circadian rhythmicity. Although many researchers sought clues as to the nature of the mechanism, little progress was made until the 1990s. Instead, during the period 1960–1990, much circadian research focused on developing more detailed accounts of circadian phenomena, such as, how the phase of circadian oscillations is affected by light pulses of different strengths and durations.

Whereas the focus on circadian rhythms was largely motivated by trying to understand the phenomenon as it is normally manifested (disruptions of circadian rhythms were largely used to identify the responsible mechanism), mood, like other psychiatric phenomena, is typically characterized in terms of disorders. The identification of *melancholia* stems from ancient times, with the term *depression* acquiring general currency by the end of the nineteenth century. DSM-I, in 1952, included the category of *depressive reaction*, and DSM-II (1968) included *depressive neurosis*. Mania, and the shifting from manic to depressive states, was also recognized in the DSM-II as manic-depressive psychosis. The former was subsequently labeled *unipolar* and the later *bipolar*. The terms *major depressive disorder* (MDD) and *bipolar disorder* (BD) were introduced in the 1970s and incorporated into DSM-III in 1980.

Suggestions of a link between mood disorders and circadian rhythms developed about the same time as the endogenous nature of circadian rhythms was established. Many of these focused on sleep disruptions in patients with mood disorders, for which there were case reports but no systematic studies until Hinton (9) performed a detailed study of currently depressed and recovered patients. He showed less sleep during each hour of the night as well as greater motility in those currently depressed. Sleep, however, is only partially under circadian control and Hinton’s, as well as a number of other studies in the 1960s and 1970s, such as, Taub and Berger’s (10) examination of the effects of altered sleep patterns on mood in healthy individuals, faced the problem of how to differentiate the effects of disrupted sleep and disrupted circadian rhythms. By employing a forced desynchrony protocol using a light–dark period longer than the circadian system could adapt to, Boivin et al. (11) were able to establish that although subjective happiness declined over each daily awake period, it clearly also oscillated in accord with underlying circadian rhythms (as measured, for example, by core body temperature).

A number of researchers in the 1970s and 1980s demonstrated correlations between measures of mood and measures of circadian rhythms. For example, Kripke et al. (12) found altered circadian periods in the manic-depressive patients they studied and determined that only those with shortened rhythms responded to lithium treatment. In another example, Souetre et al. (13) measured body temperature, plasma cortisol, norepinephrine, thyrotropin, and melatonin concentrations in depressed, recovered, and controls with no diagnosis of depression and demonstrated that the phase remained normal but the amplitude was significantly diminished in depressed participants but returned to normal after recovery.

A different strategy for establishing the linkage between mood disorders and circadian rhythms focused on various therapies found to be effective for mood disorders. Perhaps the best known is light therapy for seasonal affective disorders (SADs). After establishing that sunlight and bright artificial light (2000 lux) can suppress melatonin levels in humans, Lewy et al. (14) employed bright light therapy during 3 h after awakening on a seasonally manic-depressive patient during winter when his depression was greatest. This considerably reduced his depressive symptoms. Based on studies with additional patients, Rosenthal et al. (15) introduced the category SAD and presented further evidence of temporary reduction in depression with bright light therapy (the effects usually ceased when light treatment was stopped). The researchers went beyond the correlation to propose the *phase shift hypothesis*, which holds that depression results from the delayed phase of circadian rhythms (or, in a few cases, from an advanced phase) and that the therapeutic effect of light on depression resulted from shifting the phase of circadian rhythms earlier (or later in phase advanced patients). They supported this with evidence of advance (or delay) in the phase of melatonin expression in treated patients [(16, 17); for a more recent review, see Ref. (18)].

The evidence reviewed in this section demonstrated a connection between the phenomena of mood disorders and circadian rhythms. Mood changes according to circadian time and correlates with a number of other measures of circadian rhythmicity. Therapies that treat mood disorders, such as bright light at dawn, serve to alter the phase of circadian rhythms. The success of light therapy alleviating depression suggested a causal account, whereby circadian rhythms when altered are a causal factor in depression. Light therapy, on this account, restores normal rhythmicity and relieves depression. This idea was picked up in other theoretical proposals, such as, the *social Zeitgeber theory* (19), which proposed that in individuals vulnerable to depression, social stress events can disrupt circadian rhythms and that this in turn leads to depression. But to show that there is a direct causal connection between the phenomena requires more than evidence that they are correlated or even that the same treatments affect both. Information about the mechanism is required. Otherwise, one cannot discount the possibility that neither phenomenon directly affects the other but both phenomena are the product of other factors that are correlated or causally related.

## Research Implicating Molecular Components of the Circadian Clock in Mood Disorders

**Figure 1:**
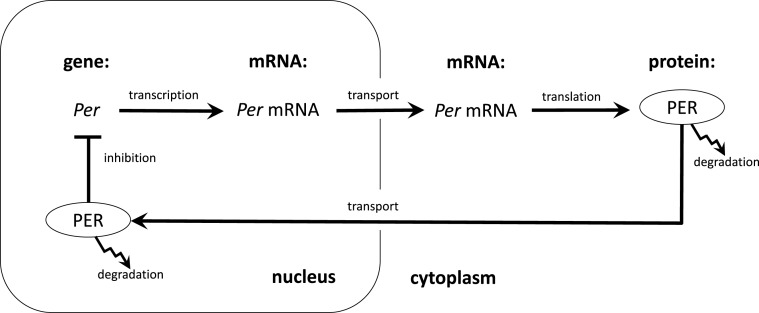
**The transcription–translation feedback loop proposed by Hardin et al. (23) to explain circadian rhythms**. Transcription of the gene *Per* and translation into protein, PER is followed by the transport of PER back to the nucleus where it inhibits the transcription of its own gene.

The initial clues to the mechanism responsible for circadian rhythms in animals were developed in the early 1970s when circadian researchers both linked the central clock mechanism in mammals to a structure in the hypothalamus known as the suprachiasmatic nucleus (SCN) (20, 21) and, in fruit flies, identified a gene, *Period* (*Per*)^2^, in which mutations caused short or long period oscillations or rendered the organisms arrhythmic (22). However, little progress was made until it was possible to clone *Per* and measure concentrations of its mRNA and protein at different times of day. This revealed that both *Per* mRNA the PER protein oscillated, with the protein peaking several hours after the mRNA. Hardin et al. (23) proposed on this basis a delayed negative feedback mechanism in which the protein PER would feedback on its own gene, temporarily inhibiting its own synthesis until concentrations decayed, at which point the inhibition would cease and new PER could be synthesized (Figure 1).

**Figure 2:**
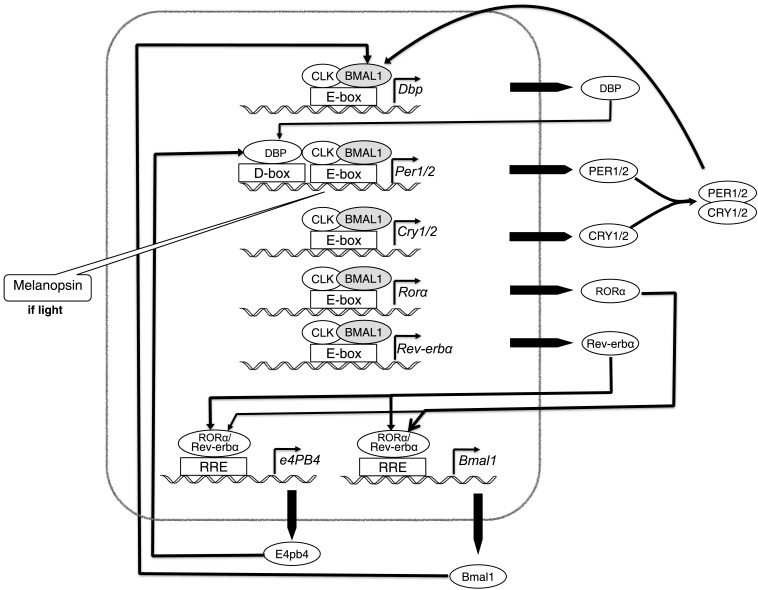
**The parts, operations, and organization of the mammalian circadian clock, as understood circa 2005**.

In the 15 years after 1990, many more clock genes were identified, including many homologs between the genes found in fruit flies and mice. Of particular, relevance for the relations between circadian rhythms and mood was the discovery of *Clock* in mice (24), which was shown to bind to the *Per* promoter and to be the target of inhibitory activity by PER. Figure 2 shows the conception of the circadian mechanism in mammalian cells that had been generated by 2005. Two variant proteins, PER1 and PER2, were now recognized as forming dimers with CRY1 and CRY2, respectively. The inhibition is directed at a dimer between CLOCK (CLK) and BMAL1, which otherwise would bind to the promoter on *Per* and several other genes, activating their transcription. In addition, there is a negative feedback loop involving *E4pb4* and a positive feedback loop involving *Bmal1*.

Much of the research on the mechanisms underlying depression has focused on several monoamine neurotransmitters, especially serotonin, but also norepinephrine and dopamine. These were identified primarily through the fact that many antidepressant drugs increase levels of these monoamines (25). Caspi et al. (26) targeted serotonin as playing a mediating role between stressful life events and depression since individuals with short alleles of serotonin transporter were more prone to experience depression after such events. The different monoamines are associated with particular brain regions that figure centrally in research on depression. The dorsal raphe nuclei are the only source of serotonin in the brain. Dopamine, which is synthesized in decreased amounts in depression, functions in projections from the ventral tegmental area (VTA) to the nucleus accumbens. Norepinephrine is expressed in the locus coeruleus.

One clue to the linkage between circadian rhythms and these neurotransmitters that are altered in depression is that serotonin, norepinephrine, and dopamine all exhibit circadian oscillations in their concentrations (27). Moreover, the connection between the circadian mechanism and synthesis of these monoamines is quite direct: *monoamine oxidase A*, *Maoa*, is a transcriptional target of clock genes *Bmal1* and *Per2* and the protein MAOA serves to terminate dopamine signaling. On the other side, several clock genes have been linked to mood disorders. *Clock*, *Bmal1*, and *Per3* have been implicated in bipolar disease. SNPs of *Per2*, *Npas2*, and *Bmal1* are linked to increased risk for SAD, while there is suggestive evidence of a link between *Cry2* and depression (28). There is also evidence suggestive of a role of mood disorders in affecting circadian rhythms. The SCN has among the densest serotonergic innervation in the brain (all five 5-HT receptor types are employed) and the innervated region of the SCN significantly overlaps areas that receive retinal input and figure in entrainment. This suggests that mood may modulate the ability of the circadian clock to be entrained to local environments, a hypothesis supported by the fact that lesioning the raphe nucleus, thereby eliminating serotonergic innervation of the SCN, alters entrainment. Moreover, applying an agonist of 5-HT receptor generates phase advances (29).

Even stronger evidence indicative of a connection between parts of the circadian mechanism and mood is found in the affects of mutant forms of clock genes on mood. Vitaterna et al.’s discovery that *Clock* is a circadian gene resulted from the generation of a mutant (*Clock*Δ19) that exhibited a long period (27 h) and arrhythmia after several days in darkness. This same mutant has provided a mouse model for mania (30). Both the mutant mice and humans with mania exhibit (1) disrupted circadian rhythms, (2) hyperactivity, and (3) decreased sleep. In addition, the mice exhibit other traits closely resembling those of mania in humans: (4) humans describe feelings of extreme euphoria, while the mice exhibit hyperhedonia and less helplessness, (5) humans engage in increased risk taking, while the mice exhibit reduced anxiety, and (6) while humans exhibit a propensity to drug abuse, the mice show increased preference for cocaine. The mutant mice exhibit increased dopamine in the VTA, rendering neurons there more excitable. As in humans, lithium normalizes manic behavior, and since one effect of lithium is to increase dopamine levels in the VTA, the link appears to be causal.

McClung and her colleagues have investigated *Clock*Δ19 mice to acquire clues into the mechanism underlying human mania. They have found deficits involving the entrainment of low gamma (30–50 Hz) oscillations to delta (1–4 Hz) oscillations in the nucleus accumbens in these mutant mice. In wild-type mice, such entrainment is negatively correlated with amount of exploration in a novel environment. This entrainment is seriously impaired in ClockΔ19 mice, which also exhibit hyperactivity in response to novelty. When treated with lithium, the coupling is restored and the hyperactivity stops (31). Knockdown of *Clock* in the VTA alone results in a manic-like state of less anxiety and hyperactivity but also depressive behavior (32), which seems to fit the fact that manic patients typically also exhibit depressive episodes. Lithium has been shown to lengthen circadian period, likely by inhibiting GSK3β, which phosphorylates PER2 and REV–ERBα, and to produce phase delays as well as affecting the amplitude and period of circadian oscillations (33). It, thus, has the opposite effects on the clock as light treatment, appropriate since it affects mania, not depression.

**Figure 3:**
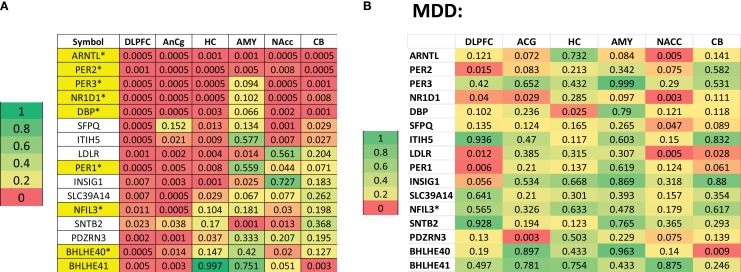
**(A)** Top oscillating genes in six brain tissues in normal controls in a cadaver study in which time of death was known. Shown in yellow are genes known to figure in the circadian clock. The numbers indicate significance levels on a measure of oscillation and color coding, with color coding differentiating five groups. **(B)** Data for patients with major depressive disorder analyzed in the same manner, making it clear that these genes achieved much lower significance levels in almost all tissues. Reproduced from Li et al. (34), Figures 2 and 4A. Copyright (2013) National Academy of Sciences, USA.

In an extremely ambitious study, Li et al. (34) provided yet additional evidence pointing to a causal link between circadian genes and mood disorders. They examined gene expression in six brain areas in cadavers of 55 normal controls and 34 patients with MDD post-mortem, analyzing them by circadian time of death. By plotting concentrations of mRNA by the patient’s circadian time of death, they generated pseudo-time series data for each gene across the subject pool. In the controls, they identified several hundred genes exhibiting cyclic expression in the dorsolateral prefrontal cortex, amygdala, cerebellum, nucleus accumbens, anterior cingulate cortex, and hippocampus. Many core clock genes were among the genes with the strongest cyclic patterns. These are labeled in yellow on the left in Figure 3, in which the *p*-values for those genes with the highest overall significance levels on a measure of oscillation are shown. Red indicates genes whose oscillation is significant (*p* < 0.05) in a given tissue. On the right are the comparable data for the patients with MDD. Comparing the two plots reveals that oscillation reaches statistical significance for many fewer genes in many fewer tissues among the patients. Especially noteworthy is the decrease of rhythmicity in ARNTL (BMAL1) and PER2, two central clock genes, in most brain tissues.

**Figure 4:**
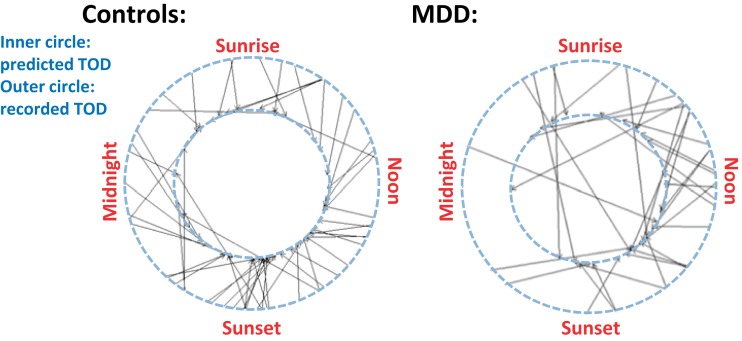
**In normal controls, as shown on the left, the pattern of gene expression at death, shown on the inner circle, was a good predictor of time of death, shown on the outer circle**. Most of the lines connect nearby points on the two circles. In depressed patients (shown on the right), however, gene expression at death provided a must less reliable predictor of time of death. Reproduced from Li et al. (34), Figure 4C. Copyright (2013) National Academy of Sciences, USA.

As a measure of the power of the post-mortem gene expression data, Li et al. used the data from 60 randomly selected subjects (both normal controls and patients) to construct an algorithm designed to predict time of death from the pattern of gene expression at death. Figure 4 shows, for all subjects, the actual time of death in the outer circle and the predicted time of death in the inner circle. Lines connect the corresponding data points and it is clear that the predictions for normal controls align better than for those for patients with MDD. This indicates substantial disruption in the circadian pattern of gene expression in the depressed patients.

Another type of evidence suggestive of a causal connection between the circadian mechanism and that involved in mood disorders is that several of the interventions designed to affect either mood or circadian rhythms also affect the other. One of the most popular drugs used to treat depression, the serotonin reuptake inhibitor (SSRI) fluoxetine, also induces phase advance in the SCN in slices of rat brain in culture. Other SSRIs shorten the circadian period. Agomelatine, a relatively recent drug that advances the phase in melatonin expression, has proven effective in treating depression (it also, though, functions as an agonist to serotonin 2C receptors so the effects might not just be through altering circadian phase).

In this section, I have discussed the development of mechanistic accounts of both circadian rhythms and mood disorders. What the research has revealed is a host of possible connections between these mechanisms and many have found these highly suggestive that circadian disruption might cause mood disorders, or vice versa. But is such evidence sufficient to establish that the mechanisms are causally linked? I turn to that question in the next section.

## How are the Mechanisms Related?

The evidence discussed in the previous sections, claiming both phenomenal and mechanistic connections between circadian rhythms and mood, invites a causal interpretation: disrupted circadian rhythms cause mood disorders (or vice versa). In fact, though, there are four different possibilities to consider:

1. Circadian disruptions cause mood disorders.
2. Mood disorders cause circadian disorders.
3. Causation goes in both directions.
4. Both circadian disruptions and mood disorders are common effects of something else or pleiotropic effects of common components.

Most interpretations adopt the first possibility. Yet, if there is a connection in that direction, there is likely also to be feedback from mood disorders to circadian disorders. However, my concern in this section is with the fourth possibility, which rejects a direct causal connection between circadian rhythms and mood disorders. I will argue that the evidence to date does not allow one to reject possibility.

One way some researchers have tried to support a causal connection is to identify intervening pathways between the two mechanisms. McClung (35) identifies several pathways by which circadian rhythms might regulate mood, of which I discuss three.

There are no direct projections from locus of the central circadian clock, the SCN, to the main loci of mood mechanisms, the dorsal raphe nucleus, the VTA, or the locus coereleus. But McClung shows that there are indirect pathways through a number of hypothalamic nuclei (e.g., the SCN projects to the dorsomedial hypothalamus which then projects to all three areas). These pathways appear to enable circadian oscillations in the SCN to regulate monoamine synthesis in these tissues. These pathways would explain how monoamine levels are altered in mutant mice in which clock genes are mutated or knocked down. McClung identifies a second pathway through the immune system. Alternations to circadian rhythms have effects on the immune system, leading to increased levels of proinflammatory cytokines. Increased levels of proinflammatory cytokines have previously been implicated in depression (36) as well as reduced neurogenesis, neural plasticity, and long-term potentiation. Moreover, the reduction in neurogenesis as well as depressive behaviors can be blocked in environments otherwise inducing stress by applying an inhibitor of nuclear factor-κB (NF-κB). This points to the NF-κB pathway as figuring in generating depression in animals with altered circadian rhythms. The determination that CLOCK itself interacts with NF-κB to activate transcription at NF-κB responsive promoters (37) further supports this as a candidate pathway for linking circadian mechanisms and mood disorders. A third pathway McClung proposed involves glucocorticoids. Concentrations of glucocorticoids increase in stress situations, a condition correlated with mood disorders. A neuronal and hormonal excitatory pathway from the SCN through the paraventricular nucleus (PVN) and the pituitary to the adrenal gland results in the rhythmic synthesis and release of glucocorticoids that then feed back onto the PVN and adrenal glands to maintain stable levels. Two clock proteins figure in regulating glucocorticoid levels. CRY proteins repress glucocorticoid receptors on the PVN and adrenal glands, thereby generating oscillation in the response to glucocorticoids. The receptors are also acetylated by CLOCK, which also decreases their sensitivity to glucocorticoids in the morning and increases it in the evening when acetylation is reversed (38).

Together with the evidence that components of the circadian clock are involved in mood, the evidence of pathways through which the circadian clock could regulate moods makes the case that the circadian clock plays a role in regulating seem plausible. But such evidence alone does not address the question as to whether the mechanisms themselves are actually linked. They may share components, but the roles these components play in each mechanism may be impendent of the role they play in the other. If that were the case, then, even if there are pathways that could connect the two mechanisms, the two mechanisms are not affecting each other – the generation of circadian rhythms is not affecting moods. Each mechanism operates on its own. Establishing that the circadian mechanism is what is affecting moods requires demonstrating that when common components contribute to one phenomenon, they do so in a way that is responsive to their role in the mechanism responsible for the other phenomenon.

Invoking the phenomenon of pleiotropy – the same gene having multiple functions – Landgraf et al. (39) make the case that the mechanisms responsible for circadian rhythms and moods may operate independently while sharing components:

it is important to point out that, although commonly called ‘clock genes’, the molecular components of the circadian clock have pleiotropic functions, including many functions that have nothing to do with the clock: manipulating clock genes affects more than just circadian rhythms.

Landgraf et al. marshal their argument by considering several of the kinds of evidence for a causal link from circadian phenomena or mechanisms to mood such as I have presented in previous sections. In response to each, they argue that the evidence is compatible with the circadian and mood mechanisms operating independently while using common components. In fact, given differences in the way the two mechanisms operate, Landgraf et al. suggest there is reason to distinguish, not integrate, the two mechanisms. I will briefly present four of their examples.

One phenomenal linkage between circadian rhythms and mood involves the use of sleep deprivation as a means of transiently ameliorating symptoms of depression in both MDD and BD patients (40, 41). Sleep deprivation has also been found to induce mania in mice (42), which could then be successfully treated with lithium or tamoxifen. Sleep deprivation has been shown as well to have effects on circadian rhythms in mice, hamsters, and humans. A possible explanation for the dual affects of sleep deprivation on both mood and circadian rhythms is that they are the product of one integrated mechanism. But Landgraf et al. note there are also important differences between the phenomena. For example, a brief nap the following day can result in a relapse of depression, but does not have any effect on the circadian system. Sleep is only partially a circadian phenomenon, and it is possible that the effects on depression depend on a non-circadian pathway, perhaps involving cytokines, cortisol, or brain-derived neurotrophic factor.

A second example involves one of the possible pathways that might link circadian disruptions and mood disorders I discussed above: the NF-κB signal transduction pathway. Monje et al. (43) have provided intriguing evidence that the immune system, specifically the NF-κB signal transduction pathway, may be a common cause of circadian disruption and mood disorders, not an intermediary. They appeal to the effects of constant darkness, a known strategy for inducing depression-like behavior in rats. It is known that total darkness results in apoptosis of noradrenergic neurons in the locus coeruleus, serotinergic neurons in the dorsal raphe, and dopaminergic neurons in the VTA. Citing evidence of the role of the immune system in depression, they show that constant darkness results in increased levels of proinflammatory cytokine IL-6 in the hippocampus, leading to increased ERK activation. Manipulation of NF-κB inhibitors indicates that NF-κB played a causal role in mood disorders. The authors also demonstrated a causal effect of NF-κB in decreasing PER2 and increasing BMAL1 levels in the hippocampus. Rather than the effect of constant darkness on mood being mediated by an effect on the circadian system, the two may be independent consequences mediated by a common immunological pathway.

I turn now to claim about two common components of the circadian and mood mechanisms: *Clock* and *Per2*. Without challenging that the appearance of manic symptoms in the *Clock*Δ19 mouse are a result of higher dopamine levels in the VTA, which are themselves the result of the *Clock* gene mutation, Landgraf et al. note that there is little evidence of endogenous circadian rhythms in the VTA (when projections from the SCN are cut, the VTA exhibits no sustained PER2:LUC rhythms). Rather, when rhythms are found in the VTA, they may be driven by the SCN. In the VTA, where *Clock* has an effect on mood, it may not be performing a circadian function at all. It might instead be an independent contribution of the same genes.

PER2 is one of the proteins most clearly oscillating in brain tissues of normal controls. As we saw in discussing Li et al.’s study, its oscillation is greatly reduced in patients with MDD. Mouse studies have shown that stress, which leads to depression-like behaviors, results in lower amplitude oscillations in PER2 (44). But the effects can be dissociated. PER2 rhythms are restored more rapidly than recovery from depression-like behaviors. The link between PER2 and mood is thought to involve MAOA, which, as discussed above, serves to terminate dopamine signaling. MAOA contains an E-box through which its transcription is made rhythmic by fluctuating PER2 and BMAL1 concentrations (45). However, one *Per2* mutant, *Per2^Brdm1−/−^*, despite no longer generating rhythmic expression of *Maoa*, still exhibits oscillation in dopamine levels in the VTA and its overall dopamine levels are higher than in the wild type. It exhibits much less depression-like behavior in response to a stressful activity like forced swim. Landgraf et al. suggest that the behavioral changes may be due to decrease in constitutive expression of dopamine, not its oscillation.

These and other examples that Landgraf et al. present illustrate a variety of ways to discount the suggestive links between the phenomena of circadian disruption and mood disorders and between the mechanisms for each. The two phenomena may be independent effects of a common cause^3^ and the responses to therapeutic intervention, while similar, may show differences to yet other manipulations. The two mechanisms may involve the same parts without the parts figuring in a common mechanism. The roles in the different mechanisms may be pleiotropic. Moreover, the mood mechanism may not be responsive to the oscillation in dopamine but to how much is expressed. Altogether these results raise doubts about whether the correlations discussed earlier between circadian disruptions and mood disorders are due to a causal linkage between the mechanisms responsible for the two phenomena.

Raising doubts about whether two phenomena or their respective mechanisms are causally connected is very different from showing that they are not. Given the variety of correlations between mood disorders and circadian disruptions, it seems highly plausible that they are causally connected. What the challenges by Landgraf et al. indicate is that different evidence is required to establish a causal link than has been provided by most of the research to date. What would seem to be required is a demonstration that perturbations that alter one phenomenon affect the other in virtue of the way they altered the first phenomenon. This might be most clearly shown in by focusing on common parts of the circadian mechanism and the mood mechanism and the way they function in each mechanism. If the effect the part has on mood depends on how it behaves in the circadian mechanism that would indicate a causal link between the mechanisms. Experimental perturbations would provide the most compelling evidence: if perturbing how a part functions within the circadian clock thereby perturbs how it behaves in the mood mechanism, then one would have strong evidence that the two mechanisms are causally integrated. Procuring such evidence will be experimentally challenging and the evidence available to date does not support such direct causal connections.

## Conclusion

Research on both circadian rhythms and mood disorders has been pursued in the quest for mechanistic explanations of each. This requires delineating the respective phenomena, linking the phenomena with a particular mechanism, and decomposing that mechanism into its parts and operations. I have reviewed several major studies that provide evidence that the phenomena of altered circadian rhythms and mood disorders are correlated and of common components in that the respective mechanisms share components. This research strongly suggests that altered circadian rhythms are a causal factor in the generation of mood disorders. Drawing on arguments advanced by Landgraf et al., however, I have argued that the evidence advanced so far does not yet justify such a claim. The phenomena may be due to common causes and the components of the mechanisms may function independently in each one. My goal, however, is not simply to make the point that correlation does not establish causation but rather to point to what would provide stronger support for the claim that circadian disruptions cause mood disorders. What is required is to show that it is as a contributor to the circadian mechanism that a component affects moods. One way this can be done is to establish that as its state in the circadian mechanism changes, the contribution of the component to the generation of moods and mood disorders changes.

The general issue of how mechanisms hypothesized to explain different phenomena are connected to one another is becoming increasingly important in biology and medicine. The investigation of proteins or the genes that code for them has often revealed components that figure in two or more cellular functions, sometimes in the same tissue. The question, as I have tried to argue here, is then whether the common component actually provides a causal linkage between the mechanisms. One factor accelerating the need to address this question is that researchers are moving beyond classical techniques that typically only identify a handful of very important components to a mechanism, such as *Per*, *Cry*, *Clock*, and *Bmal1*, in the case of circadian rhythms. Newer techniques reveal a plethora of components. For example, using small interfering RNAs to screen the whole genome, Zhang et al. (46) identified an additional 200 genes beyond those thought to constitute the core clock which have affects on the amplitude or period of circadian rhythms. Many of these genes were previously characterized in terms of their roles in other cellular functions and it becomes relevant to know whether they are operating within their role in these other cellular functions in affecting circadian rhythms. If they do, then manipulating these other phenomena may be a viable strategy for altering circadian rhythms, or vice versa.

My concern has been to present issues of this sort as arising within ongoing science. Accordingly, I have examined in detail how the issue has arisen in the case of circadian rhythm research and mood. But the issue is a general one. Finding relations between phenomena and common parts within the mechanisms proposed to explain them can be very suggestive of a causal relation. Yet, until one has shown that a component has its effect on one mechanism in virtue of its role in the other, one has not established a causal connection between the phenomena.

## Conflict of Interest Statement

The author declares that the research was conducted in the absence of any commercial or financial relationships that could be construed as a potential conflict of interest.

## Footnotes

^1^The relation between circadian rhythms and mood disorders is just one example of relations between circadian and other phenomena/mechanisms that have been the focus of intense research in recent years. Two other major examples are the relations between circadian rhythms and basic metabolism and circadian rhythms and cognition. See Venkataraman et al. (47) for a review of recent research addressing all three of these linkages between circadian rhythms and other phenomena.

^2^Conventions for naming genes differ between insects and mammals. For this paper, I will adopt the mammalian convention of italics and capitalizing the first letter in the case of genes and upper case Roman for proteins regardless of species.

^3^If one opens the investigation into common causes, one finds many possible candidates. In footnote 1, I alluded to the growing evidence of multiple points of connection between circadian mechanisms and metabolic processes. Metabolic processes are one for common causes between the circadian mechanisms and mood mechanisms.
